# Isolation of a Highly Thermostable Bile Salt Hydrolase With Broad Substrate Specificity From *Lactobacillus paragasseri*

**DOI:** 10.3389/fmicb.2022.810872

**Published:** 2022-02-17

**Authors:** Hiroyuki Kusada, Masanori Arita, Masanori Tohno, Hideyuki Tamaki

**Affiliations:** ^1^Bioproduction Research Institute, National Institute of Advanced Industrial Science and Technology, Tsukuba, Japan; ^2^Bioinformation and DDBJ Center, National Institute of Genetics, Mishima, Japan; ^3^Research Center of Genetic Resources, Core Technology Research Headquarters, National Agriculture and Food Research Organization, Tsukuba, Japan; ^4^Institute of Livestock and Grassland Science, National Agriculture and Food Research Organization, Nasushiobara, Japan; ^5^Faculty of Life and Environmental Sciences, University of Tsukuba, Tsukuba, Japan

**Keywords:** bile salt hydrolase, *Lactobacillus paragasseri*, probiotics, substrate specificity, thermostability

## Abstract

Bile salt hydrolase (BSH) enzymes produced by intestinal *Lactobacillus* species have been recognized as major targets for probiotic studies owing to their weight-loss and cholesterol-lowering effects. In this study, we isolated a highly thermostable BSH with broad substrate specificity, designed as LapBSH (BSH from a probiotic bacterium, *Lactobacillus paragasseri* JCM 5343*^T^*). The recombinant LapBSH protein clearly hydrolyzed 12 different substrates, including primary/secondary, major/minor, and taurine/glycine-conjugated bile salts in mammalian digestive tracts. Intriguingly, LapBSH further displayed a highly thermostable ability among all characterized BSH enzymes. Indeed, this enzyme retained above 80% of its optimum BSH activity even after 6 h of incubation at 50–90°C. LapBSH also exerted a functionally stable activity and maintained above 85% of its original activity after pre-heating at 85°C for 2 h. Therefore, LapBSH is a very unique probiotic enzyme with broad substrate specificity and high thermostability. The strain itself, JCM 5343^T^, was also found to exhibit high heat-resistance ability and could form colonies even after exposure to 85°C for 2 h. As thermostable enzyme/bacterium offers industrial and biotechnological advantages in terms of its productivity and stability improvements, both thermostable LapBSH and thermotolerant *L. paragasseri* JCM 5343^T^ could be promising candidates for future probiotic research.

## Introduction

Bile salts synthesized in the mammalian liver from cholesterol are surface-active and amphipathic steroid detergents that play an essential role in lipid digestion and absorption in mammalian gastrointestinal tracts. Bile salts are widely known as antimicrobial agents that inhibit bacterial growth by damaging the cell membranes of intestinal bacteria (Urdaneta and Casadesús, 2017). Some intestinal bacteria can overcome the toxicity of bile salts by producing bile salt hydrolase (BSH, EC 3.5.1.24), a key enzyme that can hydrolyze and deconjugate glycine or taurine from the cholesterol core of the bile acids (Begley et al., 2006). BSH enzymes also confer significant beneficial impacts on human health (e.g., serum cholesterol reduction) (Begley et al., 2006), in addition to their bile detoxification ability in probiotic lactic acid bacteria. A variety of intestinal bacteria have been found to exert BSH activity (Begley et al., 2006; Jones et al., 2008; O’Flaherty et al., 2018). Furthermore, *Lactobacillus* species are known to contribute to most of the total BSH activity in mammalian small intestines (Chand et al., 2017).

To date, several genes encoding BSH have been isolated from *Lacticaseibacillus casei* (Zhang et al., 2009), *Limosilactobacillus fermentum* (Kumar et al., 2013), *Lactiplantibacillus plantarum* (Christiaens et al., 1992; Lambert et al., 2008; Ren et al., 2011; Dong et al., 2012, 2013; Gu et al., 2014), *Limosilactobacillus reuteri* (Taranto et al., 1999), *Lacticaseibacillus rhamnosus* (Kaya et al., 2017), *Ligilactobacillus salivarius* (Wang et al., 2012; Bi et al., 2013), and some *Lactobacillus* spp. including *Lactobacillus acidophilus* (McAuliffe et al., 2005), *Lactobacillus gasseri* (Rani et al., 2017; Kusada et al., 2021), *Lactobacillus johnsonii* (Lundeen and Savage, 1990, 1992; Elkins et al., 2001; Oh et al., 2008; Chae et al., 2013), and *Lactobacillus paragasseri* (Kusada et al., submitted). The substrate specificity of BSH enzymes may be strain specific; however, most of the characterized BSH enzymes prefer to hydrolyze glycine-conjugated bile salts rather than taurine-conjugated ones (Dong and Lee, 2018). Furthermore, few BSH enzymes from *L. johnsonii* and *L. salivarius* have more specificity to taurine-conjugated bile salts (Oh et al., 2008; Chae et al., 2013), whereas some BSH enzymes from *L. acidophilus* and *L. plantarum* are known to hydrolyze both glycine- and taurine-conjugated bile salts (McAuliffe et al., 2005; Gu et al., 2014).

In general, most previously identified BSH enzymes function optimally under mesophilic conditions (approximately around 30–55°C) (Dong and Lee, 2018), except only one BSH enzyme from *L. johnsonii* PF01 (LjBSHC), which displays optimum activity at 70°C (Chae et al., 2013). Apart from the intestinal bacteria, a thermophilic *Brevibacillus* spp. isolated from hot spring water possesses a BSH enzyme with the optimum temperature of 60°C (Sridevi et al., 2009); however, the gene encoding the BSH enzyme has never been isolated and characterized. Thus, thermostable BSH enzyme remains largely unknown. Of note, thermostable enzyme and its thermotolerant host bacterium extend their potential application in biotechnological industries, which often require high heat treatment process, because (i) enzyme reaction at high temperature leads to improvement of productivity by reducing the viscosity of the reaction solution, increasing the solubility of substrates, and eliminating the potential risk of contamination, including pathogens (Lasa and Berenguer, 1993; Haki and Rakshit, 2003), and (ii) thermostable enzymes generally tend to be stable under harsh industrial process conditions (e.g., acids, alkalis, and organic solvents) (Lasa and Berenguer, 1993; Haki and Rakshit, 2003). Based on these biotechnological advantages, we assume that a thermostable BSH enzyme and a thermotolerant BSH-producing lactic acid bacterium could be potential new targets for future probiotic studies and could be employed in the food/pharmaceutical industries.

In the present study, we explored a new candidate of *bsh* gene from *L. paragasseri* JCM 5343^T^, a BSH-producing probiotic candidate isolated from human feces (Tanizawa et al., 2018). Intriguingly, we succeeded in discovering a novel BSH enzyme (LapBSH) with unique features, such as broad substrate specificity and highly thermostable activity among all BSHs identified, by cloning and expressing the gene from the strain JCM 5343^T^ and conducting a biochemical characterization of its enzymatic functions.

## Materials and Methods

### Bacterial Strains Used in This Study

A probiotic lactic acid bacterium *L. paragasseri* strain JCM 5343^T^ (= KCTC 3172^T^ = ATCC 4963^T^) (Tanizawa et al., 2018) was purchased from the Japan Collection of Microorganisms (JCM, RIKEN BRC, Ibaraki, Japan). Strain JCM 5343^T^ was cultivated using de Man-Rogosa-Sharpe medium (MRS, Difco Laboratories, Detroit, MI, United States) with headspace gas of N_2_/CO_2_ (80:20, *v*/*v*) at 37°C under anaerobic conditions. The *Escherichia coli* strain, DH5α (GMbiolab, Taichung, Taiwan), was used as the host strain for gene cloning, while the *E. coli* strain Origami™ 2 (DE3) (Novagen, Madison, WI, United States) was used as the host strain for the gene expression experiment. Both *E. coli* strains were cultured on Luria-Bertani (LB) agar or in LB broth (Nacalai Tesque Inc., Kyoto, Japan) supplemented with 100 μg/ml ampicillin (Sigma-Aldrich, Saint Louis, MO, United States) at 37°C.

### Cloning and Heterologous Expression of a Putative Bile Salt Hydrolase Gene

Based on the sequence analyses and homology searches using NCBI BLAST program^1^, UniProt BLAST tool^2^, InterProScan^3^, Pfam^4^, and SignalP-5.0 server^5^, a gene encoding a putative BSH was screened from the complete genome sequence of strain JCM 5343^T^ (accession number AP018549) (Tanizawa et al., 2018). A putative *bsh* gene candidate (called *lapBSH*, locus_tag = “LpgJCM5343_07940”) was amplified with PrimeSTAR HS DNA Polymerase (TaKaRa, Tokyo, Japan) using following primer set: CG GGATCCTGTACCTCAATTATTTATGATTCAAAC (*Bam*HI site underlined) and CGGAATTCATTTTGATAGTTAATA TGTTGCTTTTC (*Eco*RI site underlined). The PCR cycling program was follows: initial denaturation at 98°C for 5 min, followed by 40 cycles at 98°C for 10 s and 68°C for 60 s. Gene cloning, overexpression, and protein purification of *lapBSH* were performed according to our previous study (Kusada et al., 2017), with slight modifications. In brief, the PCR product was purified and sub-cloned into an expression vector pCold II (TaKaRa). Thereafter, the resulting plasmid was transformed into *E. coli* Origami™2 (DE3) competent cells. Isopropyl-β-D-thiogalactopyranoside (IPTG, Nacalai Tesque) was added to the culture medium at a final concentration of 100 μM when the OD_600_ of the culture was approximately 0.5. After the addition of IPTG, the *E. coli* cells were incubated overnight at 15°C with shaking. The cells were pelleted by centrifugation at 5,800 × *g* for 10 min, suspended in lysis buffer (20 mM Tris-HCl, 200 mM NaCl, 10% glycerin, pH 6.0), and disrupted by sonication using an ultrasonic disintegrator (Sonicator BRANSON Sonifer 250, Branson, Danbury, CT, United States) in an ice-water bath. The cell debris was pelleted by centrifugation, and the resulting supernatant was further purified using a standard nickel affinity chromatography method based on a previous study (Kusada et al., 2017). The purified LapBSH solution was dialyzed with semipermeable membrane (Spectra/Por 3 membrane MWCO: 3,500, Repligen, Waltham, MA, United States) at 4°C and further concentrated using VIVASPIN Turbo 4 centrifugal filter units (MWCO: 10,000 PES membrane, Sartorius, Göttingen, Germany). To verify the purity and molecular weight of a recombinant LapBSH protein, sodium dodecyl sulfate polyacrylamide gel electrophoresis (SDS-PAGE) and native-PAGE were performed using Mini-PROTEAN TGX precast polyacrylamide gel (Bio-Rad, Hercules, CA, United States). The gel was stained with QC Colloidal Coomassie Stain (Bio-Rad) *via* gentle agitation. To determine the effect of pH on oligomeric states of LapBSH protein, we used Good’s buffer solutions and adjusted the pH values (pH 3.0–10.0) of LapBSH before native-PAGE analysis.

### Bile Salt Hydrolase Activity

The BSH assay was performed using the purified LapBSH protein as described previously (Allain et al., 2018; Kusada et al., 2021). In brief, the purified LapBSH protein (100 μg/100 μl) was mixed with 0.24 mg/100 μl of the selected conjugated bile salts and incubated at 37°C. The bile salt hydrolysis reaction was terminated by adding 15% trichloroacetic acid solution (FUJIFILM Wako Pure Chemical Corporation, Osaka, Japan), and the proteins were removed by centrifugation at 10,000 × *g* for 15 min at 20°C. The supernatant was then mixed with 0.3 M borate buffer with 1% SDS (pH 9.5) and 0.3% 2,4,6-trinitrobenzenesulfonic acid solution (Tokyo Kasei Kogyo Co., Ltd., Tokyo, Japan). This mixture was statically incubated for 30 min at room temperature in the dark. Thereafter, 0.6 mM HCl was added to stop the reaction. The released glycine or taurine was measured at 416 nm using a SPARK 10M multimode microplate reader (TECAN, Männedorf, Switzerland). As a negative control, each bile salt solution was mixed with buffer instead of LapBSH protein (no enzyme control). Three independent experiments were performed (total *n* = 24). Student’s *t*-test was performed using GraphPad Prism version 8.0 software program (GraphPad Software, San Diego, CA, United States). For all analyses, a *p*-value less than 0.05 (*P* < 0.05) was defined as statistically significant. The tested substrates were glycocholic acid (GCA, Sigma-Aldrich), glycochenodeoxycholic acid (GCDCA, Sigma-Aldrich), glycodeoxycholic acid (GDCA, Sigma-Aldrich), glycoursodeoxycholic acid (GUDCA, Tokyo Kasei Kogyo), glycolithocholic acid (GLCA, Cayman Chemical, Ann Arbor, MI, United States), glycohyodeoxycholic acid (GHDCA, FUJIFILM Wako Pure Chemical Corporation), taurocholic acid (TCA, Nacalai Tesque), taurochenodeoxycholic acid (TCDCA, Sigma-Aldrich), taurodeoxycholic acid (TDCA, Nacalai Tesque), tauroursodeoxycholic acid (TUDCA, Sigma-Aldrich), taurolithocholic acid (TLCA, Cayman Chemical), and taurohyodeoxycholic acid (THDCA, FUJIFILM Wako Pure Chemical Corporation). Furthermore, we determined the effects of pre-treatment with EDTA on LapBSH activity. Briefly, the purified LapBSH protein was mixed with EDTA at a final concentration of 5 mM. After, the pre-treated LapBSH enzymes were mixed with 12 substrates, and BSH activity was detected as described above.

### Biochemical Characterization of LapBSH

Studies on the optimum enzymatic condition of LapBSH were performed as described previously (Rani et al., 2017; Kusada et al., 2021). We selected TDCA as a representative substrate and mixed it with purified LapBSH (100 μg/100 μl) at various temperatures (10–90°C, in intervals of 10°C) and pH values (pH 3.0–10.0, in intervals of pH 1.0). After 6 h of incubation, the released taurine was detected as described above. We used Good’s buffer solution to determine the effects of pH on the enzyme activity of LapBSH, acetate buffer (CH_3_COONa 3H_2_O) pH 3.0–4.0, MES buffer (C_6_H_13_NO_4_S H_2_O) pH 5.0–6.0, HEPES buffer (C_8_H_18_N_2_O_4_S) pH 7.0–8.0, and CAPS (C_9_H_19_NO_3_S) pH 9.0–10.0. In addition, the thermostability of LapBSH protein was evaluated after incubation at different temperatures (55–100°C, in intervals of 5°C) for 2 h and cooling to a room temperature. The resulting pre-heated LapBSH was mixed with TDCA for 6 h, and the released taurine was detected as described above. All experiments were performed with eight technical replicates (*n* = 8).

### Inhibitory Effects of Metal Ions on LapBSH Activity

We determined the effects of metal ions on LapBSH activity according to a previous study (Rani et al., 2017). Briefly, the purified LapBSH protein was pre-incubated with nine metal ions (CuCl_2_, CuSO_4_, MnCl_2_, MnSO_4_, MgCl_2_, MgSO_4_, ZnCl_2_, ZnSO_4_, and CaCl_2_) at a final concentration of 5 mM for 30 min at 37°C. After the pre-incubated LapBSH enzymes were mixed with TDCA and incubated at 37°C for 6 h, the released taurine was detected as described above. LapBSH protein without pre-incubation with metal ion was used as a control (no inhibition control). All experiments were performed with eight technical replicates (*n* = 8).

### Gas Chromatograph Mass Spectrometry Analysis

The penicillin acylase activity of purified LapBSH protein was demonstrated by gas chromatograph mass spectrometry (GC-MS) analysis as described previously (Kusada et al., 2017; Yasutake et al., 2017). Briefly, 10 mM of penicillin G (Nacalai Tesque) solution was mixed with the purified LapBSH and buffer as a no-enzyme control and incubated at 37°C. After incubation, the digestion mixtures were extracted three times with equal volumes of ethyl acetate (FUJIFILM Wako Pure Chemical Corporation). Thereafter, the organic phase was evaporated to dryness in vacuum for 10 min (EYELA, Tokyo, Japan). The resulting sample was re-dissolved in methanol (FUJIFILM Wako Pure Chemical Corporation) and introduced onto a SHIMADZU GCMS-QP5050 system (Shimadzu Co., Ltd., Kyoto, Japan) equipped with a DB-5 capillary column (30 m × 0.25 mm, 0.25-μm film thickness; Agilent Technologies, Palo Alto, CA, United States).

### Conserved Amino Acid Sequence Analysis

A multiple-amino-acid sequence alignment analysis was performed using the CLUSTAL W2 program and GENETYX-MAC software version 20.1.1 (GENETYX, Tokyo, Japan). The amino acid sequence of LapBSH was aligned with that of characterized BSHs from lactic acid bacteria.

### Thermal Resistance Test of *Lactobacillus paragasseri*

Strain JCM 5343^T^ was cultured in MRS broth at 37°C under anaerobic conditions. Full-grown cultures were heat-treated at temperatures ranging from 50°C to 75°C with different incubation times according to the Food Sanitation Law in Japan. After heat treatment, the cultures were streaked on MRS agar with or without 0.25% TDCA and incubated anaerobically at 37°C. The JCM 5343^T^ culture was also heat-treated at 85°C for 30–150 min (in intervals of 30 min), and the resulting cultures were spotted on MRS agar plates and cultivated at 37°C under anaerobic conditions. *L. gasseri* JCM 1131^T^ and *L. helveticus* JCM 1004 obtained from the JCM were cultured and heat-treated as described above to serve as heat-sensitive controls. Moreover, the number of viable colony-forming-units (CFU) was compared before and after heat-treatment at 50–80°C (in intervals of 10°C) for 30 min. All experiments were performed with three technical replicates (*n* = 3).

## Results and Discussion

### Sequence Analyses of a Putative Bile Salt Hydrolase Gene

Based on sequence analyses and homology searches, we found a putative BSH gene (called *lapBSH*) in the *L. paragasseri* JCM 5343^T^ genome (Supplementary Figure 1). To obtain a recombinant LapBSH protein, we constructed a heterologous *lapBSH* gene expression system using *E. coli* Origami™ 2 (DE3) and purified the His-tagged LapBSH protein by nickel affinity chromatography. Based on SDS-PAGE analysis, the molecular weight of purified LapBSH protein was approximately 40.0 kDa in size (Supplementary Figure 2A), which is almost consistent with its calculated molecular mass based on the amino acid sequence of LapBSH (326 amino acids).

The amino acid sequence of LapBSH showed a significantly high sequence similarity with previously identified BSH (LgBSH) from *L. gasseri* FR4 (99.39%) (Rani et al., 2017), followed by BSHs from *L. johnsonii* strains (84.38 and 84.06%) and *L. acidophilus* strains (62.50 and 60.82%). LapBSH further exhibited moderate similarity to BSHs from *L. plantarum* strains (52.22 and 51.90%) and *L. salivarius* strains (45.17–47.66%). Interestingly, LapBSH showed a relatively low sequence similarity (39.32%) with LpBSH, the other BSH isolated from the same host strain, JCM 5343^T^ (Kusada et al., submitted). These sequence analyses suggest that LapBSH may be a potential BSH enzyme; however, its substrate specificity and biochemical characteristics remain unclear since biochemical features of BSHs are often in discord with their high sequence similarities (Lundeen and Savage, 1990, 1992; Elkins et al., 2001; Oh et al., 2008). Accordingly, we attempted to identify and compare the enzymatic characteristics of LapBSH with experimentally identified BSHs, especially in LgBSH and thermostable BSHs.

### Bile Salt Hydrolase Activity of a Recombinant LapBSH

**FIGURE 1:**
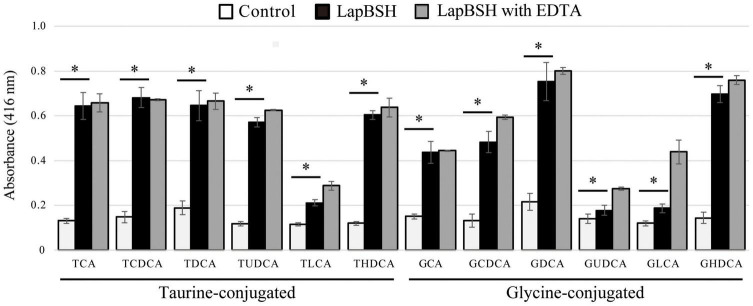
Bile salt hydrolase (BSH) activity and substrate specificity of LapBSH. The tested substrates were glycocholic acid (GCA), glycochenodeoxycholic acid (GCDCA), glycodeoxycholic acid (GDCA), glycoursodeoxycholic acid (GUDCA), glycolithocholic acid (GLCA), glycohyodeoxycholic acid (GHDCA), taurocholic acid (TCA), taurochenodeoxycholic acid (TCDCA), taurodeoxycholic acid (TDCA), tauroursodeoxycholic acid (TUDCA), taurolithocholic acid (TLCA), and taurohyodeoxycholic acid (THDCA). Values represent the mean of three independent experiments (each *n* = 8). Error bars represent standard deviation (SD). *A *p*-value less than 0.05 (*P* < 0.05) was defined as statistically significant.

To determine the BSH activity and substrate specificity of LapBSH, we used 12 different mammalian bile salts, including glycine-conjugated (GCA, GCDCA, GDCA, GUDCA, GLCA, and GHDCA) and taurine-conjugated (TCA, TCDCA, TDCA, TUDCA, TLCA, and THDCA) bile salts, by detecting the release of glycine or taurine from the hydrolysis of conjugated bile salts. As shown in Figure 1, LapBSH exhibited a notable hydrolysis activity toward all conjugated bile salts tested. In particular, this enzyme displayed high hydrolyzing activity toward six human major conjugated bile salts (TCA, TCDCA, TDCA, GCA, GCDCA, and GDCA), which constitute almost all of the conjugated bile salts in human intestine, and three minor conjugated bile salts (GHDCA, TUDCA, and THDCA) (Figure 1). LapBSH also showed lower but significant BSH activity toward other three minor substrates (TLCA, GUDCA, and GLCA) (Figure 1). A very small amount of GHDCA, TUDCA, GUDCA, TLCA, and GLCA have been found in humans (Sacquet et al., 1983; Mims and Hercules, 2003; Moreira et al., 2017; Semba et al., 2017), whereas THDCA has been absent in humans but present in rodents (Schmidt et al., 2019). Such findings suggest that LapBSH has broad substrate specificity toward minor as well as major bile salts. Therefore, LapBSH could act as a functional BSH enzyme and provide ecological advantages related to the bile detoxification for the host, *L. paragasseri* JCM 5343^T^, to enhance its survivability in human intestine. Of note, we confirmed the almost equal or slightly increased LapBSH activity when pre-treated with 5 mM EDTA (Figure 1), owing to the removal of metal ions (BSH inhibitors) in the buffers.

To date, many previously characterized BSHs from lactic acid bacteria (e.g., *Bifidobacterium* spp. and *Lactobacillus* spp.) have been reported to hydrolyze six major human-conjugated bile salts (TCA, TCDCA, TDCA, GCA, GCDCA, and GDCA) as well as LapBSH. However, the hydrolysis activity of these identified BSHs toward the residual six minor bile salts (TUDCA, TLCA, THDCA, GUDCA, GLCA, and GHDCA) has not been fully revealed. However, three known BSHs (BlBSH from *B. longum* and LjBSHA and LjBSHC from *L. johnsonii*) can hydrolyze TUDCA and THDCA, while LapBSH was capable of hydrolyzing all of them. Besides, LgBSH sharing high sequence similarity with LapBSH could hydrolyze four major bile salts (GCA, GDCA TCA, and TDCA) (Rani et al., 2017); however, its hydrolytic activity toward other residual substrates (TCDCA, TUDCA, TLCA, THDCA, GCDCA GUDCA, GLCA, and GHDCA) is unknown. Furthermore, LgBSH prefers to hydrolyze glycine-conjugated bile salts rather than taurine-conjugated bile salts (Rani et al., 2017), while LapBSH can hydrolyze both glycine- and taurine-conjugated bile salts, indicating that LapBSH and LgBSH have different substrate specificities, regardless of their high sequence similarities. Therefore, LapBSH is a new BSH enzyme with broad substrate specificity that can deconjugate 12 different bile salts, including primary/secondary, major/minor, and taurine/glycine-conjugated bile salts in mammalian digestive tracts.

### Penicillin Acylase Activity of LapBSH

In addition to BSH activity, we further determined whether recombinant LapBSH could hydrolyze β-lactam antibiotics, as some previously identified BSHs also showed penicillin acylase activity and the capability of hydrolyzing penicillin (Rossocha et al., 2005; Chand et al., 2017; Rani et al., 2017). For verification, GC-MS analyses were performed to detect phenylacetic acid generated *via* the hydrolysis of penicillin G according to our previous study (Kusada et al., 2017). We observed a significant peak with GC retention time of 5.299 min after the recombinant LapBSH was mixed with penicillin G solution (Supplementary Figure 3A). Furthermore, mass spectrometry of the 5.299-min GC fraction revealed the M-H ion at *m/z* 136 (Supplementary Figure 3B), aligning with the molecular weight of phenylacetic acid. Of note, this significant peak was not detected when penicillin G solution was incubated with buffer (data not shown). These findings clearly indicate that recombinant LapBSH also exhibits penicillin acylase activity as well as BSH activity and further emphasize its broad substrate specificity. It is noteworthy that LgBSH also exhibited penicillin acylase activity as well as LapBSH (Rani et al., 2017), suggesting that the amino acid residue(s) involved in their bi-functionality (BSH and penicillin acylase) would be conserved in these enzymes.

### Biochemical Characterization of LapBSH

**FIGURE 2:**
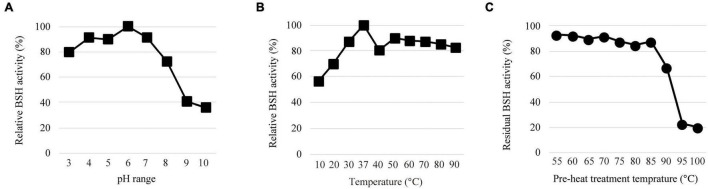
Biochemical features of LapBSH. **(A)** The effect of pH (pH 3.0–pH 10.0), **(B)** temperature (10°–90°C), and **(C)** pre-heat treatment (55°–100°C) on BSH activity toward TDCA. Each value represents the mean of eight technical replicates (*n* = 8). Maximum activity was recorded as 100%. Error bars indicate SD.

We determined the effects of pH and temperature on the enzyme activity of LapBSH as described in the experimental procedures. The maximum BSH activity of LapBSH was observed at pH 6.0 (Figure 2A). LapBSH exhibited a stable activity and retained above 80% of its original activity at pH 3.0–7.0; however, a significant decrease in enzyme activities was observed at pH values higher than 8.0. As for optimum temperature, the maximum BSH activity of LapBSH was observed at 37°C (Figure 2B). More importantly, LapBSH retained above 80% of its residual activity in high temperature ranges of 50–90°C (Figure 2B), strongly suggesting that LapBSH could have high thermostability. Indeed, LapBSH was functionally stable with above 85 and 65% retention of its original activity when the proteins were pre-heated at 85°C and 90°C for 2 h, respectively (Figure 2C).

Of note, LgBSH, the most closely related enzyme to LapBSH, displayed optimal activity at pH 5.5 and 52°C, and enzyme activity significantly decreased with increasing temperatures (e.g., approximately 50 and 10% of its original activity at 60°C and 70°C, respectively) (Rani et al., 2017). Such finding indicates that LgBSH has little or no thermostability, and the biochemical characteristics of LapBSH and LgBSH markedly differed despite a sequence similarity above 99%. We further compared the thermostable activity of LapBSH with two experimentally identified thermostable BSHs. Sridevi et al. (2009) reported that the BSH from *Brevibacillus* sp. was active in the temperature range from 30°C to 80°C (optimum at 60°C); however, its residual BSH activity decreased by about 35% of its original activity at 70°C after 1 h of pre-incubation (Sridevi et al., 2009). Chae et al. (2013) also demonstrated that the optimum activity of BSH from *L. johnsonii* PF01 (LjBSHC) occurred at 70°C, and its residual BSH activity at 90°C decreased below 20% of its original activity (Chae et al., 2013).

To clarify whether LapBSH was denatured by heat treatment, native-PAGE analysis of LapBSH was performed. As shown in Supplementary Figure 2B, we confirmed obvious bands of LapBSH proteins after heat treatment at 90°C for up to 120 min, suggesting that a part of LapBSH proteins remain at 90°C. Furthermore, based on the native-PAGE analysis, the molecular weight of native LapBSH was approximately 160 kDa in size (Supplementary Figure 2B), which corresponded to its tetramer. We further confirmed that oligomerization states of LapBSH were affected under acidic (pH 3.0 and 4.0) and alkaline pH conditions (pH 9.0 and 10.0) (Supplementary Figure 2C), suggesting that pH condition contributes to electrostatic interaction at the interface between subunits of LapBSH. Xu et al. (2016) reported that two BSH molecules (derived from *L. salivarius*) packed as a dimer in the asymmetric unit (Xu et al., 2016). This suggests that the presence of a higher assembly form (e.g., tetramer assembled as a pair of dimers) may cause a large shift in the stability of LapBSH, because oligomerization of proteins may enhance the cooperative interaction between subunits and lead to increased stability against heat denaturation (Goodsell and Olson, 2000). Maes et al. (1999) reported that some other thermophilic enzymes exist as higher-order association states compared with their mesophilic analogues. In fact, triosephosphate isomerases (TIM) from three hyperthermophilic microorganisms (*Thermotoga maritima*, *Pyrococcus woesei*, and *Methanothermus fervidus*) are known to form tetrameric assemblies (Kohlhoff et al., 1996; Bell et al., 1998; Maes et al., 1999), whereas all the other known bacterial and eukaryotic TIMs form dimeric complexes (Kohlhoff et al., 1996). Furthermore, Villeret et al. (1998) reported that heat stabilization of the ornithine carbamoyltransferase (OTCase) dodecamer in *Pyrococcus furiosus* mainly results from hydrophobic interfaces between four trimers, while other mesophilic OTCases are homotrimeric (Villeret et al., 1998). Based on these previous studies, we assume that a higher assembly form accounts for the thermostability of LapBSH. Furthermore, a future study regarding a thermal denaturation curve of LapBSH could reveal a better understanding of its reversibility and cooperativity of stabilizing factors contributing to the high stability. Thus, to the best of our knowledge, LapBSH is a highly thermostable BSH among all of the previously identified BSH enzymes.

### Inhibitory Assay of LapBSH Activity

**FIGURE 3:**
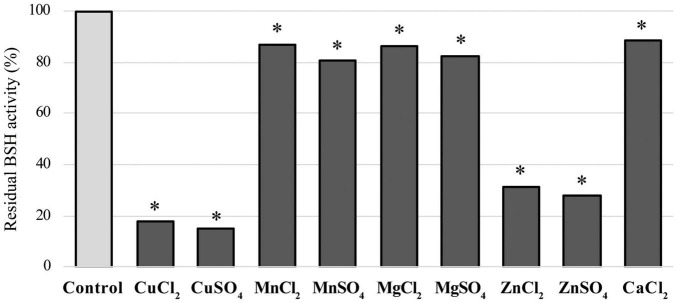
Effects of ion compounds on LapBSH activity. LapBSH proteins were pre-incubated with each of the nine metal ions at a final concentration of 5 mM for 30 min at 37°C. BSH activities were measured based on the activity of the pre-incubated LapBSH enzymes toward TDCA. Values represent the mean of eight technical replicates (*n* = 8). Maximum activity was recorded as 100%. Error bars indicate SD. *A *p*-value less than 0.05 (*P* < 0.05) was defined as statistically significant.

We determined the effects of nine metal ions (CuCl_2_, CuSO_4_, MnCl_2_, MnSO_4_, MgCl_2_, MgSO_4_, ZnCl_2_, ZnSO_4_, and CaCl_2_) on LapBSH activity. These compounds were reported to inhibit BSH activity (Rani et al., 2017), though their inhibition mechanisms are largely unknown. Among these chemicals tested, copper compounds (CuCl_2_ and CuSO_4_) and zinc compounds (ZnCl_2_ and ZnSO_4_) were found to inhibit more than 80 and 65% of LapBSH activity, respectively (Figure 3). Furthermore, other manganese, magnesium, and calcium compounds were found to display a lower inhibitory activity (approximately 11–20%) of LapBSH (Figure 3). Interestingly, although LapBSH has a high sequence similarity with LgBSH from *L. gasseri* FR4 (Rani et al., 2017), the inhibitory effects of metal ions on the BSH activity of these two BSHs were also significantly different. In fact, manganese compounds (MnCl_2_ and MnSO_4_) significantly inhibited LgBSH (>70% inhibition) but did not notably inhibit LapBSH (<20% inhibition) activities. In contrast, ZnSO_4_ showed relatively low (<40% inhibition) and high (>70% inhibition) inhibitory activities toward LgBSH and LapBSH, respectively (Rani et al., 2017). It has also been found that MnCl_2_ and MnSO_4_ clearly inhibited the enzyme activity (68.1 and 83.1%, respectively) of BSH from *L. salivarius* NRRL B-30514 (Wang et al., 2012). Furthermore, Tanaka et al. (2000) demonstrated that MgSO_4_ and CaCl_2_ significantly inhibited the enzyme activity of BSH from *Bifidobacterium longum* SBT2928 (84 and 94% inhibition, respectively) (Tanaka et al., 2000), while MgSO_4_ and CaCl_2_ rarely inhibited the BSH activity of LapBSH (17.41 and 11.58%, respectively). Finally, a strong enzyme inhibition (95.2%) of BSH from *L. plantarum* CK102 in the presence of CaCl_2_ has been reported (Ha et al., 2006). Based on the findings of previous studies and this study, the effects of metal ion inhibitors on BSH activity may vary among BSHs.

### Multiple Sequence Alignment Analysis

**FIGURE 4:**
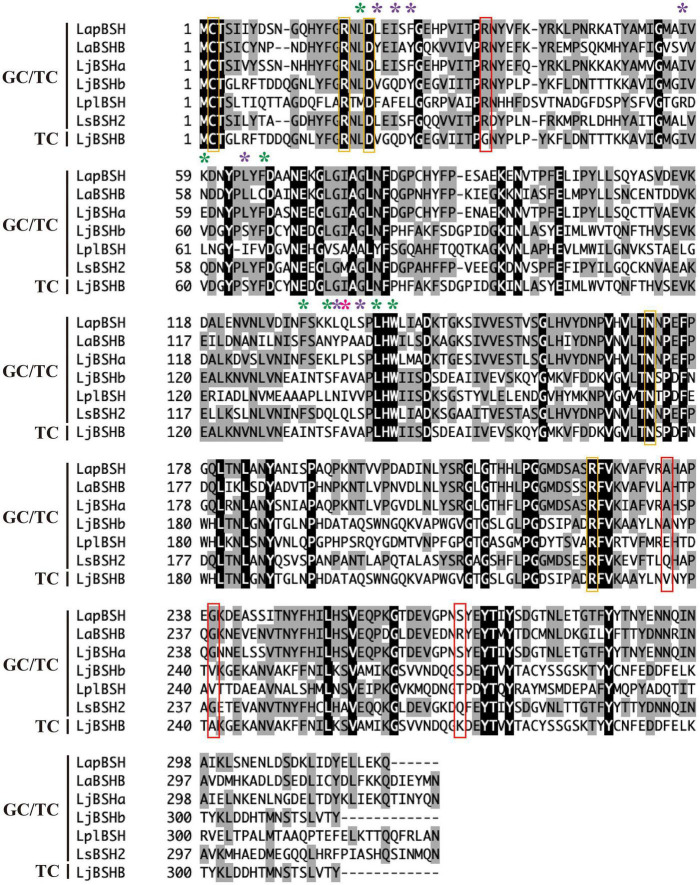
Multiple alignment of the amino acid sequences of LapBSH and other BSHs from *Lactobacillus* species. The black shading and gray shading represent identical and similar amino acid residues, respectively. The conserved residues (Cys, Arg, Asp, and Asn) associated with the predicted active site are indicated in orange boxes. Amino acid residues involved in the potential substrate specificity of known BSHs (CpBSH, BlBSH, and LgBSH) are indicated by green, purple, and pink asterisks, respectively (Rossocha et al., 2005; Kumar et al., 2006; Rani et al., 2017; Dong and Lee, 2018). Four amino acid residues that are not conserved in LjBSHB (TC) are indicated in red boxes. Abbreviated as follows: LapBSH (BBD48441), LaBSHB (AAV42923), LjBSHa (AAG22541), LjBSHb (AAC34381), LplBSH (AGG13404), LsBSH2 (ACL98205), and LjBSHB (EF536029).

The amino acid sequence of LapBSH was aligned with five BSHs known to hydrolyze both glycine- and taurine-conjugated bile salts (GC/TC) and one BSH that can hydrolyze only taurine-conjugated bile salts (TC) (Figure 4). The five residues (Cys-2, Arg-17, Asp-20, Asn-172, and Arg-225 in LapBSH) associated with active site and catalytic reaction were completely conserved in all proteins (Figure 4) (Dong and Lee, 2018). Among these conserved residues, N-terminal cysteine residue (Cys-2) is commonly known to play a critical role as a catalytic nucleophile in this protein family (Rossocha et al., 2005). However, seven amino acid residues (Leu-19, Lsy-59, Phe-66, Phe-130, Lys-133, Leu-138, and Trp-140 in LapBSH; indicated by green asterisk) that are involved in the potential substrate specificity (Rossocha et al., 2005; Dong and Lee, 2018) were not fully conserved among the proteins (Figure 4). Furthermore, another seven amino acid residues (Leu-21, Ile-23, Phe-25, Ile-57, Leu-64, Leu-133, and Ser-136 in LapBSH; indicated by purple asterisk) (Kumar et al., 2006; Dong and Lee, 2018) and one residue (Gln-134 in LapBSH; indicated by pink asterisk) (Rani et al., 2017; Dong and Lee, 2018) responsible for the putative substrate specificity were not fully conserved (Figure 4). These results suggest the presence of different amino acid residue(s) involved in substrate specificity.

The substrate preference of BSH enzymes vary among *Lactobacillus* strains of the same species. Indeed, LjBSHb from *L. johnsonii* 100-100 hydrolyzes both GC and TC (Lundeen and Savage, 1990, 1992; Elkins et al., 2001), while LjBSHB from *L. johnsonii* PF01 hydrolyzes only TC (Oh et al., 2008). However, very interestingly, these two BSHs (LjBSHb and LjBSHB) show very high amino acid sequence similarity (98.73%), and only four amino acid residues are substituted (Supplementary Figure 4A). Therefore, we assume that these four amino acid residues may be related to the substrate specificity. Indeed, we discovered that four residues of LjBSHB (TC) were not conserved in other BSHs (GC/TC), including LapBSH. In particular, Gly-35 of LjBSHB was completely substituted by Arg in other BSHs (GC/TC) (Figure 4), inferring that these residues may possibly be associated with the potential substrate preference of BSH enzymes.

LapBSH showed a high sequence similarity (99.39%) to LgBSH from *L. gasseri* FR4 (Rani et al., 2017), and only two amino acid substitutions (M97V and S178G) were confirmed (Supplementary Figure 4B). Based on the phylogenetic analysis, LapBSH and LgBSH were confirmed to be closely related with each other and were clearly categorized into the same BSH subgroup (Supplementary Figure 5). However, LapBSH and LgBSH differ mostly in terms of substrate specificity, inhibitory effects of metal ions on BSH activity, optimum temperature, and thermostability as described above (see Supplementary Table 1). Wright (1991) reported that asparagine and glutamine residues are susceptible to deamidation related to protein breakdown and aging in high-temperature environments, and this deamidation reaction would be facilitated by serine residues within the same protein (Wright, 1991). Anbar et al. (2010) demonstrated that a single mutation (S329G) dramatically increased the thermostability of endoglucanase in a thermophilic anaerobic bacterium, *Clostridium thermocellum* (Anbar et al., 2010). However, two mutations (M97V and S178G) may not be the only reason for the overall heat stabilization in LapBSH: changes of the Gibbs free energy and melting temperature by point substitutions are usually not large. In addition to two mutations, we speculate that the oligomerization of LapBSH is involved in its thermostability due to an increase in the cooperative interaction between two subunits, leading to structural stabilization as mentioned above. To clarify this point, future crystallographic study and site-directed mutagenesis analysis of LapBSH would enable a further understanding of its thermostability.

### Thermotolerance Ability of *Lactobacillus paragasseri* JCM 5343^T^

**FIGURE 5:**
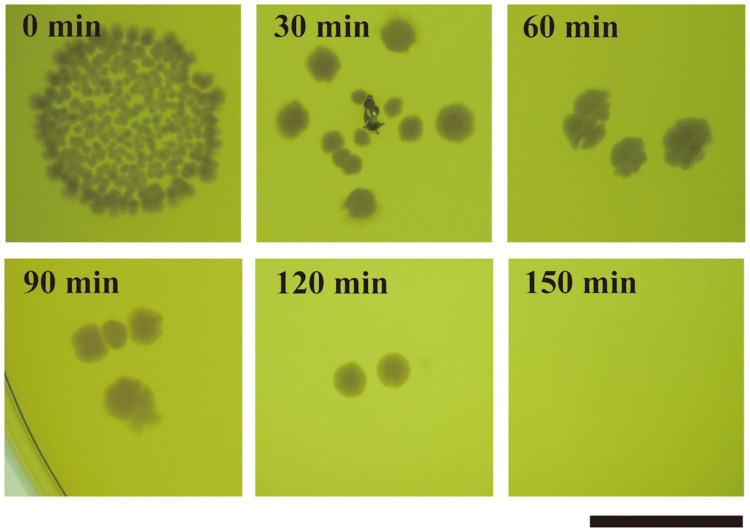
Thermal resistance assay of *L. paragasseri* JCM 5343^T^. Strain JCM 5343^T^ culture was heat treated at 85°C for 30–150 min (intervals of 30 min). After heat treatment, the resulting cultures were spotted on de Man-Rogosa-Sharpe medium (MRS) agar plates and incubated at 37°C under anaerobic conditions. Scale bar indicates 1 cm.

We determined whether strain JCM 5343^T^ has thermotolerance ability. According to the Food Sanitation Law in Japan, full-grown cultures of strain JCM 5343^T^ were heat treated at different temperatures (50–75°C) for different incubation times (57 s to 5 h). We confirmed that strain JCM 5343^T^ could grow on MRS agar under all culture conditions tested (Supplementary Figure 6), although members of the *Lactobacillus* genera are generally known to be heat sensitive. Of note, BSH activity and bile salt resistance ability of strain JCM 5343^T^ were maintained after exposure to all heat-treatment conditions (Supplementary Figure 6). We further determined the effects of heat treatment on *L. paragasseri* JCM 5343^T^ by CFU counting. The mean CFU/milliliter in the starting sample was (8.8 ± 0.5) × 10^9^. After heat treatment at 50°C and 60°C, the CFU decreased slightly to (3.4 ± 0.4) × 10^9^ and (5.8 ± 1.1) × 10^7^, respectively. After heat treatment at 70°C and 80°C, the CFU was largely decreased (Supplementary Table 2). Surprisingly, strain JCM 5343^T^ could form colonies reproducibly after heat treatment at 85°C for 120 min (Figure 5), though only a few colonies survived each time. As the other two well-known probiotic *Lactobacillus* species [*L. gasseri* JCM 1131^T^ (Kusada et al., 2021) and *L. helveticus* JCM 1004 (Pan et al., 2005)] were strictly heat sensitive and could not form colonies after heat treatment at 85°C (Supplementary Figure 7), *L. paragasseri* JCM 5343^T^ may have higher thermostability than other probiotic *Lactobacillus* species.

As mentioned above, we demonstrated that not only LapBSH but also its host microorganism (*L. paragasseri* JCM 5343^T^) retained high thermostability. The thermostable LapBSH enzyme found in the present study may largely contribute to the BSH activity and bile salt resistance ability of strain JCM 5343^T^ under heat-treatment conditions, as strain JCM 5343^T^ has the other BSH enzyme (LpBSH) found in our previous study; this BSH enzyme displayed BSH activity only under mesophilic conditions (up to 40°C) and had a significant reduction in enzyme activities at temperatures higher than 50°C (Kusada et al., submitted). The extension of the industrial application of both thermostable LapBSH and its thermotolerant host *L. paragasseri* JCM 5343^T^ would be of great advantage in oppressive biotechnological processes, including high temperature, acidic/alkaline pH, and organic solvents. A further investigation is needed to verify whether LapBSH/JCM 5343^T^ can function and maintain their probiotic effects under high-temperature conditions *in vivo* and *in vitro*.

## Conclusion

In the present study, we discovered a novel BSH enzyme (LapBSH) from *L. paragasseri* JCM 5343^T^ and verified its two unique enzymatic traits. The first unique feature of the LapBSH enzyme is its broad substrate specificity. Indeed, our extended analysis demonstrated that the LapBSH enzyme could hydrolyze up to 12 different conjugated bile salts, including primary/secondary bile salts (e.g., GCA and GDCA), taurine/glycine-conjugated bile salts (e.g., TCA and GCA), and major/minor bile salts in human intestine (e.g., GCA and GHDCA). This enzymatic feature could confer bile-detoxification ability on the host bacterium, *L. paragasseri* JCM 5343^T^, to enhance its survivability in mammalian intestine. The other outstanding feature of LapBSH is thermostability. Although few thermostable BSH enzymes have been reported to date, LapBSH exhibited markedly higher thermostability than the other BSHs identified. Furthermore, we demonstrated that strain JCM 5343^T^ displayed high heat-resistance ability and could grow after exposure to 85°C for 2 h, although *Lactobacillus* species are known to display heat susceptibility. Taken together, our findings suggest that both LapBSH and *L. paragasseri* JCM 5343^T^ could be unprecedented candidates for future probiotic research, as thermostable enzyme and thermotolerant bacterium have significant industrial and biotechnological advantages for improving enzymatic productivity and stability relative to their mesophilic counterparts.

## Data Availability Statement

The original contributions presented in the study are included in the article/Supplementary Material, further inquiries can be directed to the corresponding authors.

## Author Contributions

HK, MA, MT, and HT made significant contributions in the conceptualization and investigation. HK and HT performed the data curation, formal analysis, funding acquisition, supervision, and writing—original draft. HT was the project administrator. All authors contributed to the article and approved the submitted version.

## Conflict of Interest

The authors declare that the research was conducted in the absence of any commercial or financial relationships that could be construed as a potential conflict of interest.

## Publisher’s Note

All claims expressed in this article are solely those of the authors and do not necessarily represent those of their affiliated organizations, or those of the publisher, the editors and the reviewers. Any product that may be evaluated in this article, or claim that may be made by its manufacturer, is not guaranteed or endorsed by the publisher.

## Footnotes

1
http://www.ncbi.nlm.nih.gov/

2
https://www.uniprot.org/blast/

3
http://www.ebi.ac.uk/interpro/search/sequence-search

4
http://pfam.xfam.org/

5
http://www.cbs.dtu.dk/services/SignalP/
