# Supplementary material for: Isolation of a Highly Thermostable Bile Salt Hydrolase With Broad Substrate Specificity From Lactobacillus paragasseri
Source: Front Microbiol. 2022 Feb 17;13:810872. doi: 10.3389/fmicb.2022.810872 (PMC8893165; doi:10.3389/fmicb.2022.810872)
Supplement: Supplementary file 1 [file Data_Sheet_1.docx]

Supplementary Material


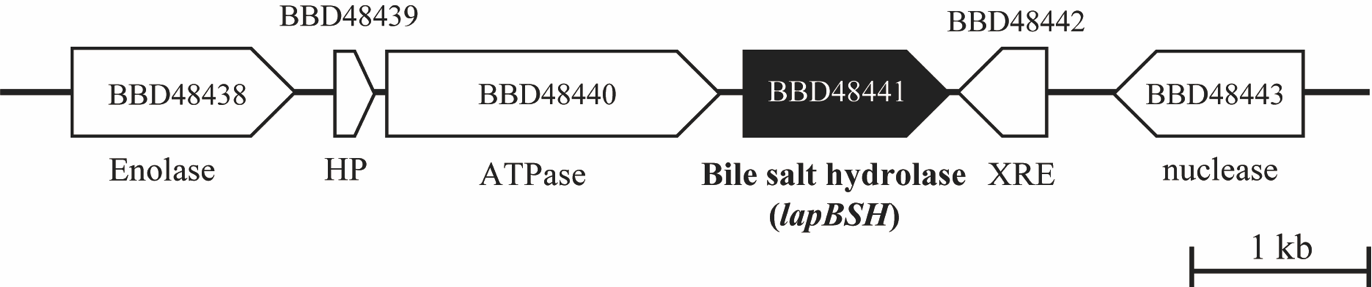


**Supplementary Figure 1.** Physical map of a predicted bile salt hydrolase (*bsh*) gene on the genome sequence of *L. paragasseri* JCM 5343^T^ (accession number AP018549). The scale bar indicates a 1 kb length of nucleotide. A putative *bsh* gene and other ORFs are represented by filled and open symbols, respectively. Protein ID and brief annotation were provided. HP and XRE indicate hypothetical protein and XRE family transcriptional regulator, respectively.


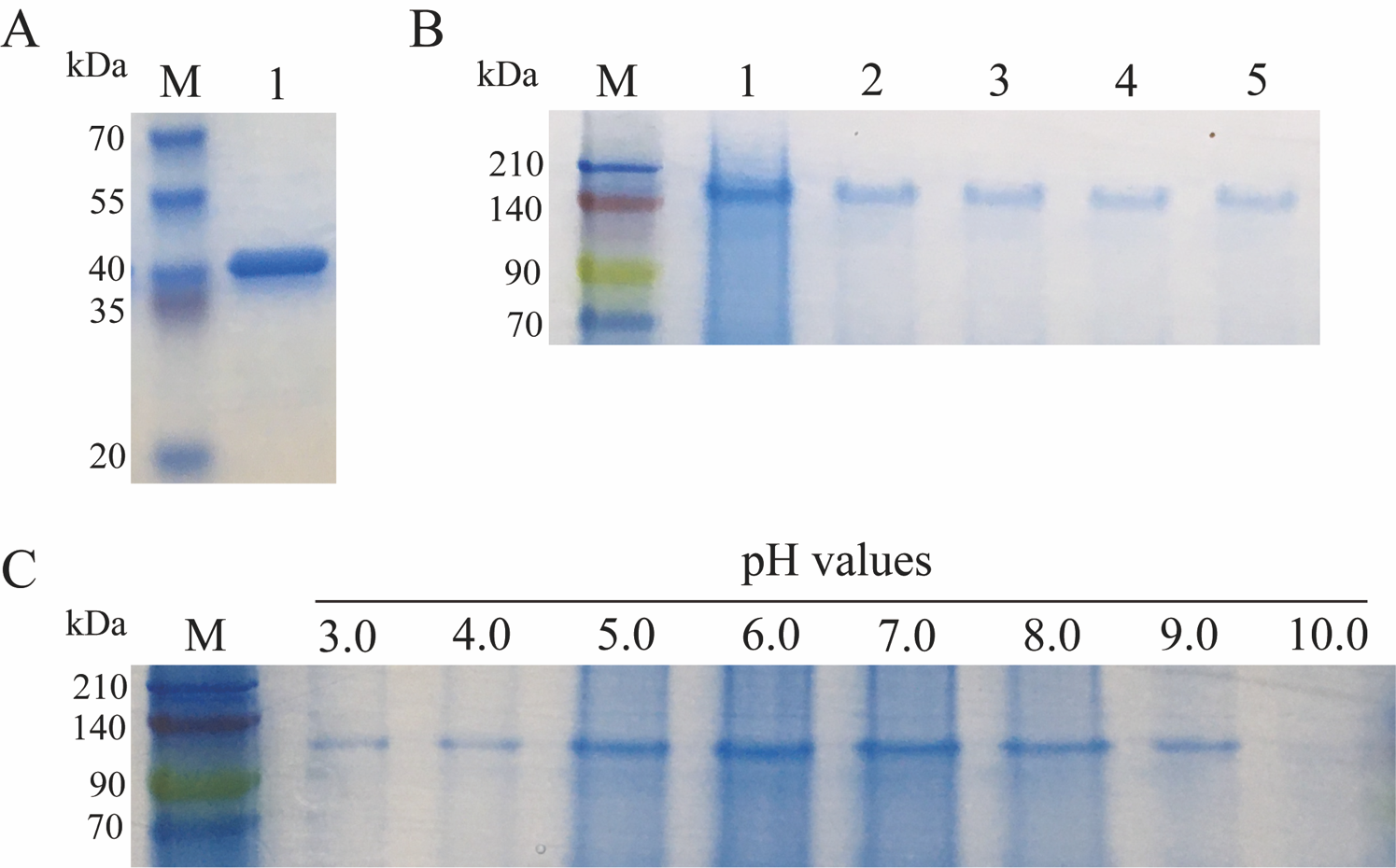


**Supplementary Figure 2.** SDS-PAGE (A) and Native-PAGE (B) analyses of purified LapBSH. After nickel affinity chromatography, purified LapBSH protein was loaded on 4-20% Mini-Protean TGX Pre-cast polyacrylamide gel (Biorad). Lane M, molecular size-marker (3-Color Prestained XL-Ladder, APRO Science); lane 1, purified LapBSH protein; lane 2-5, LapBSH proteins after heat-treatment at 90°C for 30-120 min (intervals of 30 min). Native-PAGE analysis of LapBSH with different pH values (C).


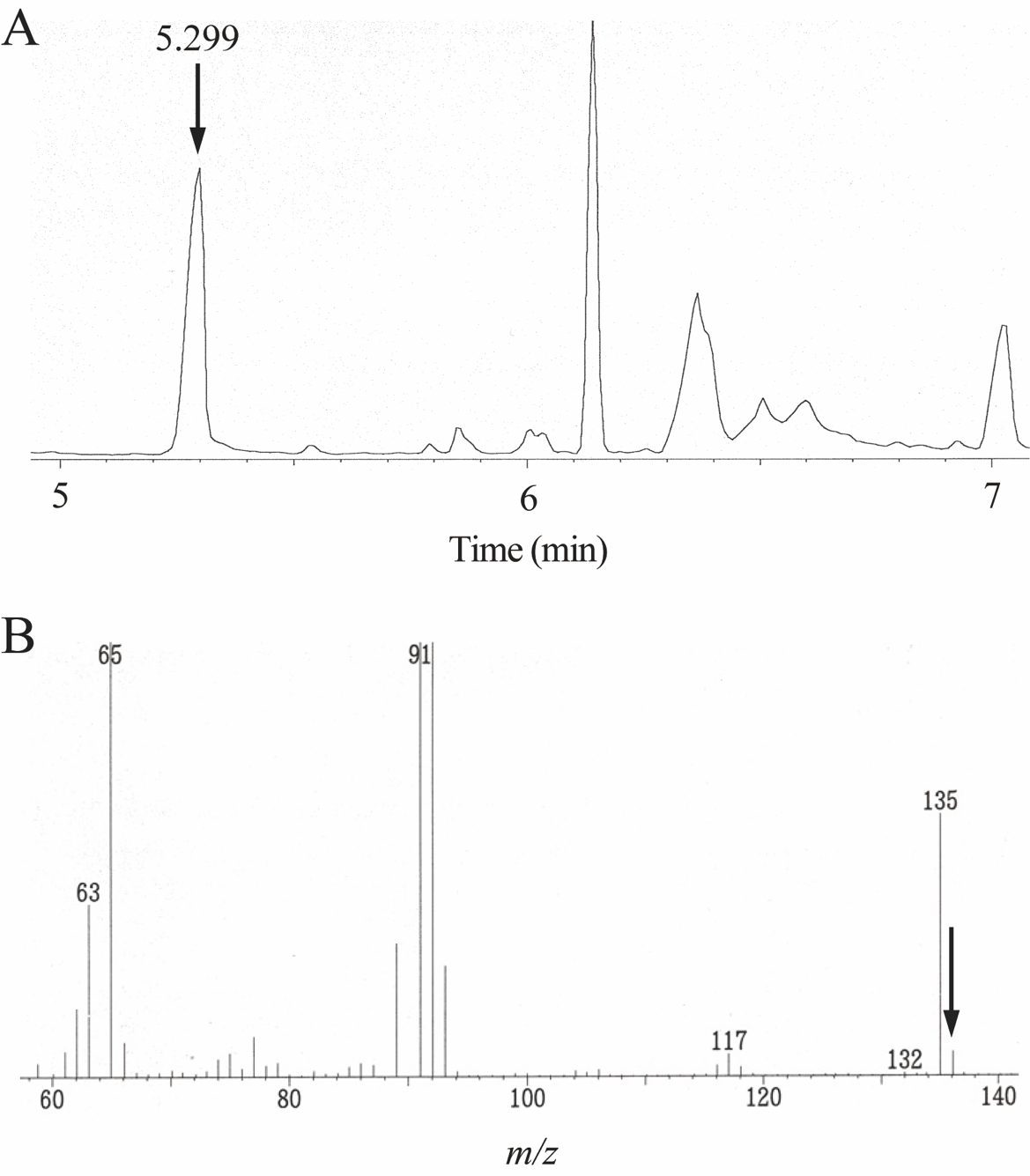


**Supplementary Figure 3.** GC-MS analysis of penicillin G degradation products by LapBSH. (A) Gas chromatography profile of digestion product of penicillin G by LapBSH. (B) Mass spectrometry of retention time at 5.299 min.


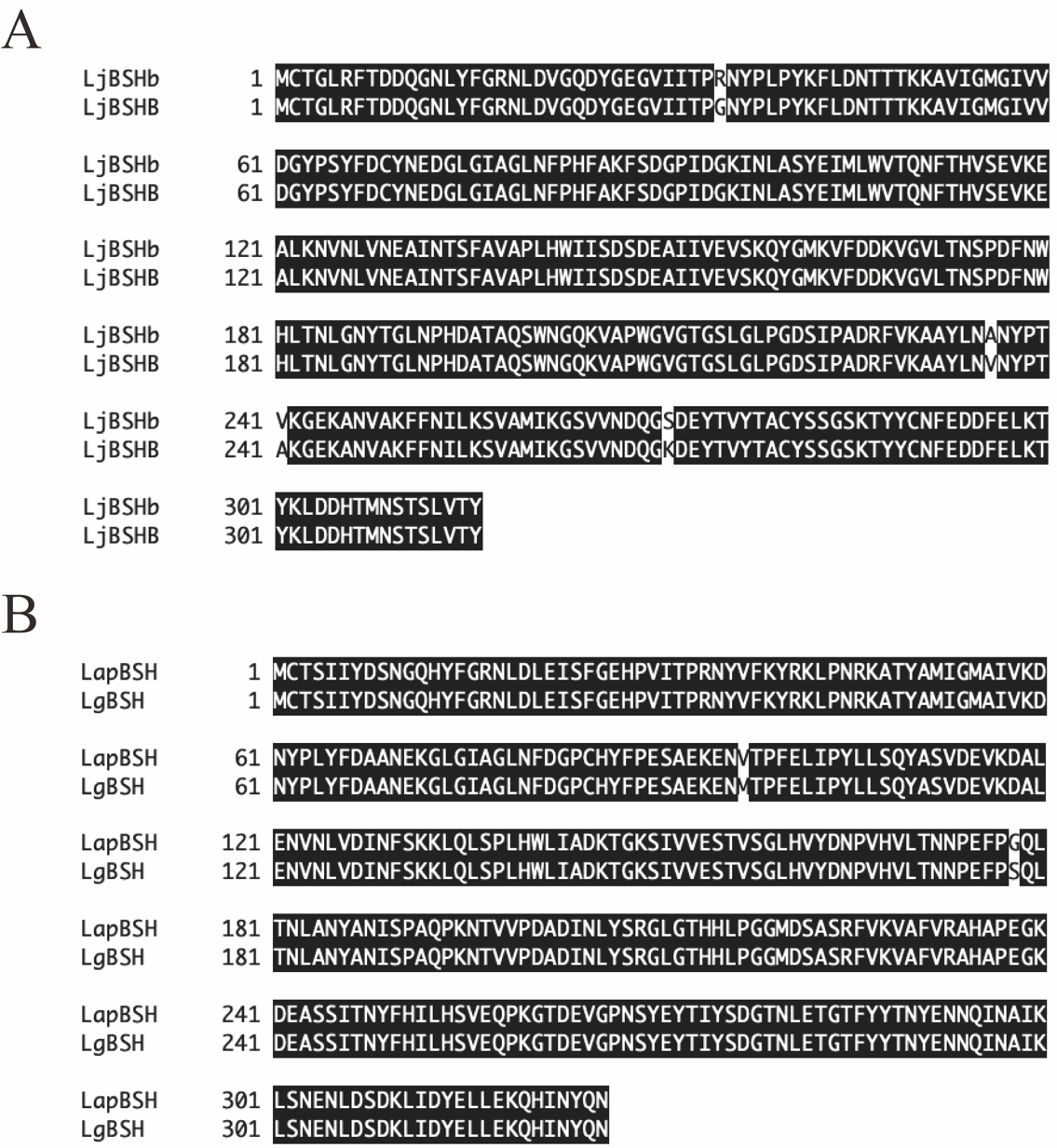


**Supplementary Figure 4.** Multiple alignment of amino acid sequences of (A) LjBSHb (AAC34381) and LjBSHB (EF536029), and (B) LapBSH (BBD48441) and LgBSH (WP_020806888).


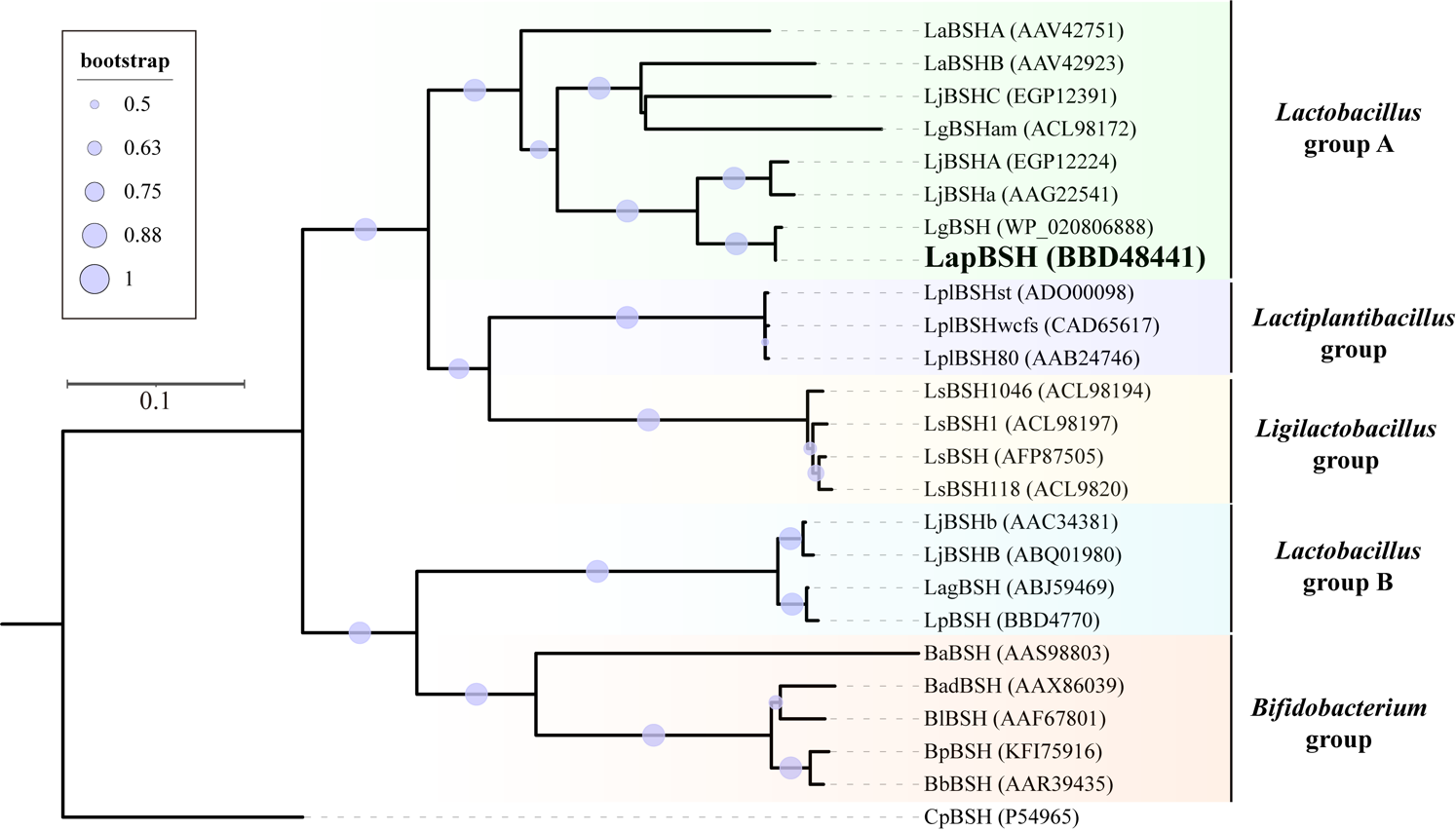


**Supplementary Figure 5.** Phylogenetic analysis of LapBSH with experimentally identified BSHs. Amino acid sequences of experimentally identified BSH proteins were used in this analysis (accession numbers were provided). The phylogenetic tree was constructed with MEGA X software using the neighbor joining method (1,000 bootstrap replications). The phylogenetic tree was displayed and customized using an online tool Interactive Tree Of Life (iTOL v5). Bootstrap values greater than 50% based on 1,000 replications are shown by circle symbols whose size correlates with the bootstrap values. CpBSH, BSH from *Clostridium perfringens* 13 was used as an outgroup.


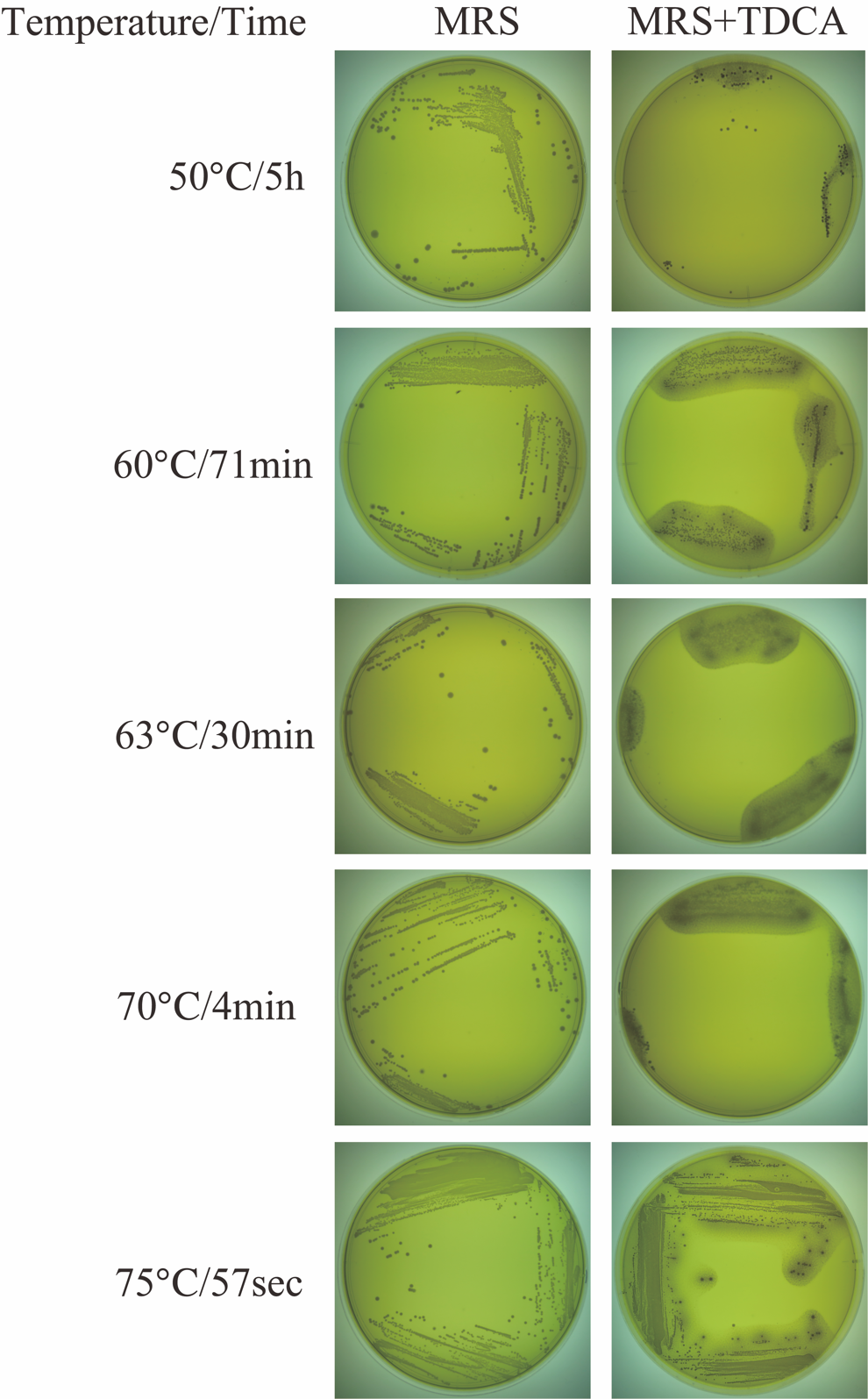


**Supplementary Figure 6.** Thermal resistance test of strain JCM 5343^T^. The full-grown cultures ware heat-treated at different temperatures ranging from 50°C to 75°C with different incubation times (57 seconds to 5 hours) according to the Food Sanitation Law in Japan. After heat treating, the cultures were streaked on MRS agar (left) and MRS agar with 0.25% TDCA (right). All agar plates were incubated at 37°C under anaerobic condition.


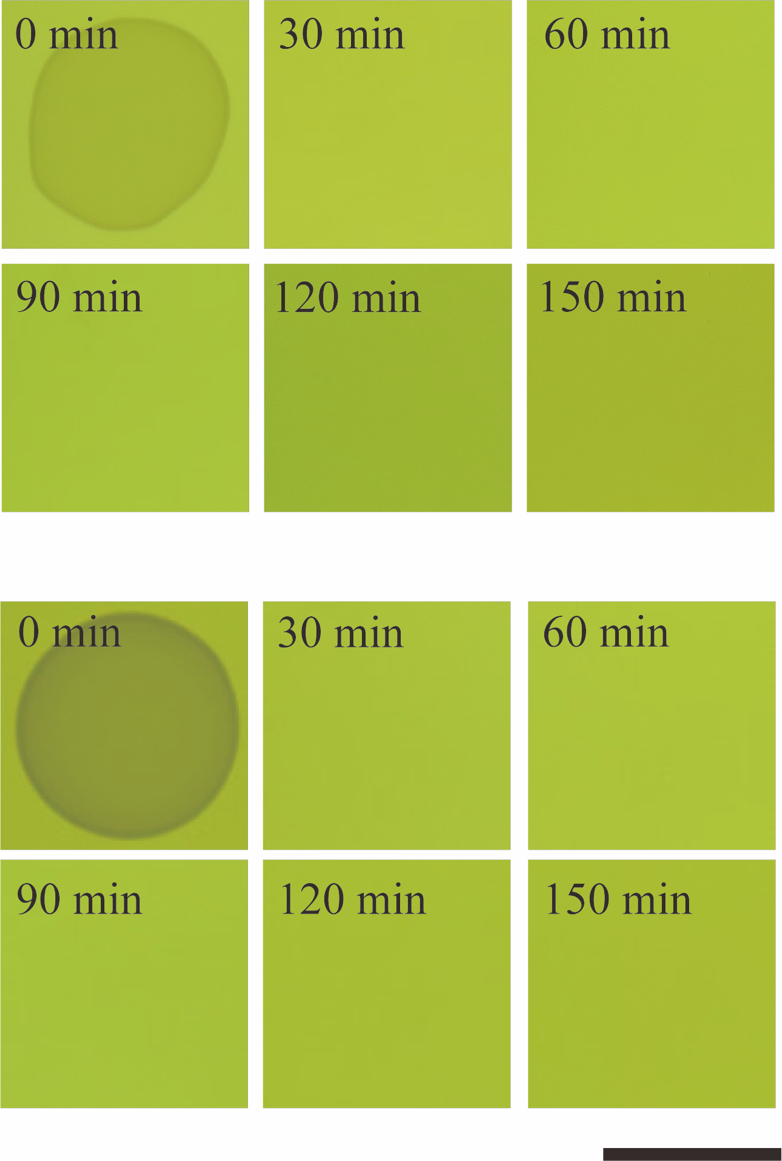


**Supplementary Figure 7.** Thermal resistance assay of *L. gasseri* JCM1131^T^ (top) and *L. helveticus* JCM1004 (bottom). Full-grown cultures were heat-treated at 85°C for 30-150 min with intervals of 30 min. After heat treating, the resulting cultures were spotted on MRS agar plates and incubated at 37°C under anaerobic condition. Scale bar indicates 1 cm.

**Supplementary Table 1.** Comparison of enzymatic characteristics between LapBSH and LgBSH.

|  | LapBSH (this study) | LgBSH (Rani et al, 2017) |
| --- | --- | --- |
| Sequence similarity | 99.39% | |
| Substrate preference | GC/TC | GC |
| Degradable substrates | GCA, GCDCA, GDCA, GUDCA, GLCA, GHDCA TCA, TCDCA, TDCA, TUDCA, TLCA THDCA | GCA, GDCA, TCA, TDCA |
| Penicillin acylase activity | Yes | Yes |
| Optimum temperature | 37℃ | 52℃ |
| Termostability (*e.g*., residual BSH activity at 70℃) | about 87.6%  of optimum activity | about 10%  of optimum activity |
| Optimum pH | 6.0 | 5.5 |
| BSH inhibitor (*e.g*., >70% inhibition) | CuCl_2_, CuSO_4_, MnCl_2_, MnSO_4_ | CuCl_2_, CuSO_4_, ZnCl_2_, ZnSO_4_ |

**Supplementary Table 2.** Colony-forming-units with or without heat-treatment for 30 min.

|  | Heat-treatment (℃) for 30 min | | | | |
| --- | --- | --- | --- | --- | --- |
|  | Control (no heat-treatment) | 50℃ | 60℃ | 70℃ | 80℃ |
| CFU/ml | (8.8 ± 0.5) × 10^9^ | (3.4 ± 0.4) × 10^9^ | (5.8 ± 1.1) × 10^7^ | (7.5 ± 0.8) × 10^4^ | (3.9 ± 0.4) × 10^4^ |
